# SARS-CoV2 infection: functional and morphological cardiopulmonary changes in elite handball players

**DOI:** 10.1038/s41598-021-97120-x

**Published:** 2021-09-07

**Authors:** S. Fikenzer, A. Kogel, C. Pietsch, D. Lavall, St. Stöbe, U. Rudolph, U. Laufs, P. Hepp, A. Hagendorff

**Affiliations:** 1 Klinik und Poliklinik für Kardiologie, Universitätsklinikum Leipzig, Liebigstr. 20, 04103 Leipzig, Germany; 2 Institut für Medizinische Mikrobiologie und Virologie - Fachbereich Virologie, Universitätsklinikum Leipzig, Johannisallee 30, 04103 Leipzig, Germany; 3 Klinik und Poliklinik für Orthopädie, Unfallchirurgie und Plastische Chirurgie, Universitätsklinikum, Leipzig, Germany

**Keywords:** Cardiology, Health care

## Abstract

There is increasing evidence of cardiac involvement post-SARS-CoV-2 infections in symptomatic as well as in oligo- and asymptomatic athletes. This study aimed to characterize the possible early effects of SARS-CoV-2 infections on myocardial morphology and cardiopulmonary function in athletes. Eight male elite handball players (27 ± 3.5 y) with past SARS-CoV-2 infection were compared with four uninfected teammates (22 ± 2.6 y). Infected athletes were examined 19 ± 7 days after the first positive PCR test. Echocardiographic assessment of the global longitudinal strain under resting conditions was not significantly changed (− 17.7% vs. − 18.1%). However, magnetic resonance imaging showed minor signs of acute inflammation/oedema in all infected athletes (T2-mapping: + 4.1 ms, *p* = 0.034) without reaching the Lake-Louis criteria. Spiroergometric analysis showed a significant reduction in VO2max (− 292 ml/min, − 7.0%), oxygen pulse (− 2.4 ml/beat, − 10.4%), and respiratory minute volume (VE) (− 18.9 l/min, − 13.8%) in athletes with a history of SARS-CoV2 infection (*p* < 0.05, respectively). The parameters were unchanged in the uninfected teammates. SARS-CoV2 infection caused impairment of cardiopulmonary performance during physical effort in elite athletes. It seems reasonable to screen athletes after SARS-CoV2 infection with spiroergometry to identify performance limitations and to guide the return to competition.

## Introduction

The global coronavirus disease 2019 (COVID-19) pandemic caused by severe acute respiratory syndrome coronavirus type 2 (SARS-CoV-2) affected all age groups, including young and physically active individuals. There is growing evidence in the literature of cardiac involvement in symptomatic as well as in asymptomatic individuals after SARS-CoV-2 infections^1^. Elite athletes may be at an increased risk of sudden cardiac death due to myocarditis during SARS-CoV-2 infections because of their overall higher physical activity^2,3^. Therefore, outbreaks in professional teams represent a potential risk for all athletes involved. To this date, a validated screening strategy for myocardial effects of SARS-CoV-2 in elite athletes is missing. Specifically, the usefulness of spiroergometry for the evaluation post COVID-19 has not been assessed^4^.

A recent MRI study on 26 athletes with and without symptoms reported 4 cases with signs of myocarditis. Eight athletes showed late enhancement in MRI without an elevation in T2 weighting^5^. The functional significance of these imaging findings is still unknown. Athletes with persistent symptoms for weeks and months after SARS-CoV-2 infection are reported in the literature^6^. Additionally, a cohort study of 1597 athletes of the Big Ten American football conference identified 37 athletes (2.3%) with signs of clinical or subclinical myocarditis^4^. Cardiac MRI is very sensitive, but not readily available for screening of athletes all over the world. Therefore, the need for additional data on screening strategies is still high.

Data on the impact of SARS-CoV-2 infection on cardiopulmonary performance are sparse. This information is important for a safe return to sport and the athletes’ long-term health^7–9^. The study aims to characterize the possible cardiac involvement of a SARS-CoV-2 infection in athletes. For this purpose, we evaluated parameters of spiroergometry, as well as echocardiography and magnetic resonance imaging (MRI). To our knowledge, this is the first study to combine functional and image morphological data in the screening of a homogeneous group of athletes after SARS-CoV-2 infection.

In addition, we obtained information on the practical aspects of spiroergometry in individuals post SARS-CoV-2 infection and examined the potential risk for clinical personnel by performing RT-PCR analysis of swab samples taken from the spirometry masks to assess a possible residual excretion of virus particles.

## Methods

### Subjects

Eight male elite handball players from the first division in Germany with SARS-CoV-2 infection and four noninfected teammates presenting for the yearly pre-season medical check-up were enrolled at the cardiological outpatient clinic and were retrospectively analyzed. The infected athletes were all part of one cluster outbreak. The characteristics of the participants are shown in Table 1. In our study group, seven athletes had a mild illness according to international guidelines^10^, one remained asymptomatic. The study was conducted in accordance with the Declaration of Helsinki on Ethical Principles and was approved by the Ethical Committee of the Medical Faculty, University of Leipzig (reference number 036/21-ek). Informed consent was obtained from all the participating athletes. Each subject was examined during the summer 2020 (t0) preparation phase and either after SARS-CoV-2 infection in winter 2020 or during routine control in winter 2020 (t1).

**Table 1:** Clinical information, cardiac magnetic resonance imaging, and quantitative echocardiographic data from all athletes included in this study.

Clinical parameter	Mean (SD)	Mean (SD)	*p*
	COVID-19	Non-COVID-19	
N	8	4	
Age,y	27 (3.5)	22 (2.6)	
Weight, kg	96.7 (5.4)	96.0 (8.0)	ns
Height, cm	190.8 (5.4)	193.5 (10.4)	ns
Time from the positive COVID-19 test result to clinical tests, d	19 (7)		
**Clinical symptoms**	Yes	No	
No symptoms, No	1		
Headache, No	5		
Rhinitis, No	4		
No sense of taste and/or smell, No	3		
Chest pain, No	2		
Breathing problems, No	2		
Sore throat, cough, sniffles, No	1		
Listlessness, No	1		
Aching limbs, No	1		
ECG-abnormalities rest, No	0	0	
ECG-abnormalities exercise, No	1	0	
**Echocardiographic measurements**			
Global longitudinal strain (GLS), %	− 18.1 (1.8)	− 17.4 (1.7)	ns
Pericardial effusion, No	1	0	
**Cardiac MRI measurements (left ventricular)**			
Ejection fraction, %	58.3 (4.7)	58.3 (4.1)	ns
End-diastolic volume index, ml/m2	60.9 (4.8)	61.1 (6.0)	ns
End-systolic volume index, ml/m2	25.6 (4.1)	25.5 (3.0)	ns
Stroke volume index, ml/m2	35.4 (3.2)	35.6 (4.6)	ns
Native T1 value, ms (reference: 1250 ms)	1205 (55)	1215 (24)	ns
Base	1202 (53)	1216 (41)	ns
Middle	1203 (61)	1205 (27)	ns
Apex	1210 (54	1222 (11)	ns
Number of segments above reference	2.6 (3.5)	2.5 (3.7)	ns
Native T2 value, ms (reference: 45 ms)	49.1 (4.4)	48.7 (0.8)	ns
Base	47.8 (4.0)	48.2 (2.8)	ns
Middle	48.5 (3.5)	49.4 (1.1)	ns
Apex	51.2 (4.4)	48.7 (2.0)	ns
Number of segments above reference	13.6 (3.5)	13.3 (4.0)	ns
Late gadolinium enhancement present, No	2	0	

*ECG* Electrocardiogram, *ns* Not significant, *No* Number.

### Study design

**Figure 1:**
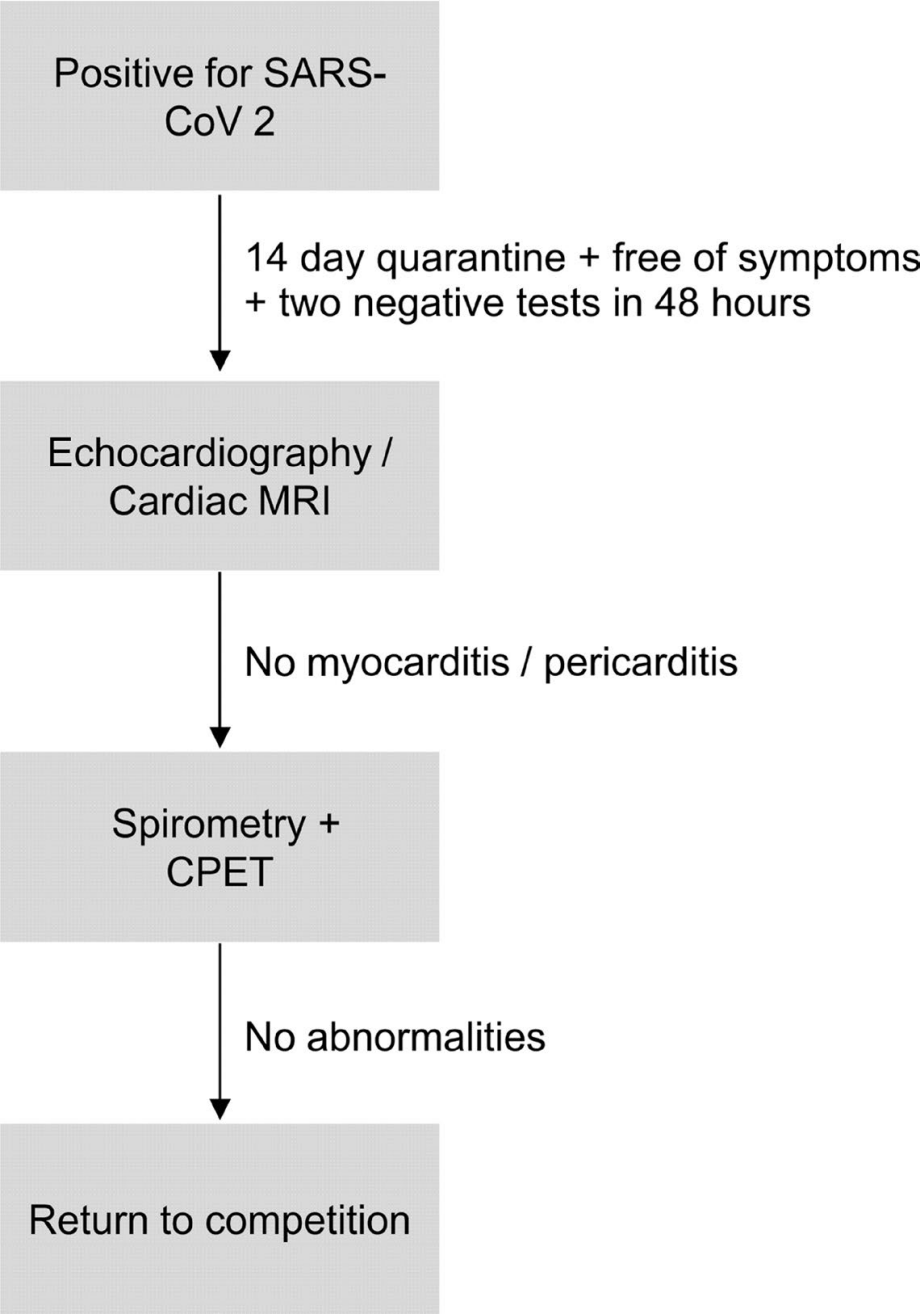
Clinical procedure after SARS-CoV-2 infection in elite athletes. In athletes who did not reach the criteria for proceeding to the next step, the diagnostic procedure was repeated after 2 weeks.

Athletes had to be tested negative for SARS-CoV-2 by specific real-time RT-PCR in nasopharyngeal swabs two times in 48 h before the examinations. Before the first test, all athletes were under quarantine for 14 days and had to be free of symptoms. The uninfected teammates did not participate in structured team training for at least two weeks, because due to high numbers of SARS-CoV-2 infections in the general population team training was disbanded. In the COVID-19 group, medical history and symptoms were taken using a questionnaire. Subjects received a physical examination and vital parameters, body measurements, and a resting electrocardiogram (ECG). Each patient was examined via MRI and transthoracic echocardiography. When no signs of myocarditis were found based on fulfilling Lake-Louis criteria, incremental cardiopulmonary exertion tests (CPET) were performed. Respiratory function was quantified using spirometry (Fig. 1).

### Echocardiographic examination

TTE was performed as described earlier^11^ using a Vivid E9 or E95 ultrasound system with an M5-S or 6-VT phased array probe (GE Healthcare Vingmed Ultrasound AS, Horten, Norway). Echocardiographic analyses were performed with the EchoPac software (Version 204, GE Healthcare Vingmed Ultrasound AS, https://www.gehealthcare.com/products/ultrasound/vivid/echopac, Horten, Norway) using the quantitative analysis software package. Global (GLS) longitudinal deformation was assessed by speckle tracking in all standardized apical views. The endocardial contour was manually adjusted, whereas only segments with accurate tracking by careful visual evaluation were accepted to exclude imaging artifacts. Tracking areas were adjusted to enable full myocardial tracking, excluding epicardial as well as valvular or atrial structures.

### Magnetic resonance imaging (MRI)

A comprehensive cardiac magnetic resonance imaging (CMR) examination was performed, including steady-state free precession cine sequences, T1 and T2 mapping, T2STIR (T2 weighted imaging with short tau inversion recovery), early and late gadolinium enhancement (LGE) using a T1 weighted fast gradient-echo sequence with adjusted inversion recovery pre-pulse as well as phase-sensitive inversion recovery sequence, on a 3.0-T scanner (Achieva, Philips Medical System, Best, The Netherlands) with a 32-channel phased-array surface coil with dS anterior and posterior coil. A standardized protocol was used as described earlier^12^. Cine images were acquired covering the entire left ventricle in contiguous short axis and long axis using a steady-state free precession sequence (slice thickness 8 mm, no interslice gap). Quantitative analyses were performed using Philips IntelliSpacePortal software(Version 11.1, Philips, https://www.philips.de/healthcare/product/HC881103/intellispace-portal11 Amsterdam, The Netherlands).

### Incremental cardiopulmonary exertion tests (CPET)

CPET was performed on a semi-recumbent ergometer (GE eBike, GE Healthcare GmbH, Solingen, Germany) at a constant speed of 60–70 revolutions per minute (rpm). The test began at a workload of 50 W with an increase of 50 W every 3 min until volitional exhaustion occurred. Each subject continued for an additional 5-min recovery period at a workload of 25 W. In the CPET, spirometry data were collected using a digital spirometer (Vyntus™ CPX, Vyaire Germany, Hoechberg, Germany). Maximum oxygen consumption (VO2max), minute ventilation (VE), and HR (GE-Cardiosoft, GE Healthcare GmbH, Solingen, Germany) were monitored continuously at rest, during IET, and during recovery. After IET the masks were sampled by swiping the inside surface with swabs to evaluate possible residual infectious risk.

### Detection and characterisation of SARS-CoV-2 viral genomes

Viral RNA in oropharyngeal and mask swabs was assessed by real-time RT-PCR (Alinity m SARS-CoV-2 assay, Abbott Molecular, Des Plaines, IL, USA) following the manufacturer’s instruction. In samples with sufficient viral RNA (Ct value ≥ 30) viral whole-genome sequences were obtained using the EasySeq RC-PCR SARS CoV-2 kit (NimaGen B.V., Nijmegen, The Netherlands) and a NextSeq sequencing system (Illumina, San Diego, CA, USA). Viral sequences were aligned and evaluated using Geneious Prime software (Version 2019.2. Biomatters, https://www.geneious.com/ Auckland, New Zealand). Viral lineages were assessed by Pangolin COVID-19 Lineage Assigner^13^.

### Statistical analysis

All values are expressed as means and standard deviations unless otherwise stated, and the significance level was defined as *p* < 0.05. Data were analyzed using Microsoft Office Excel® 2010 for Windows (Microsoft Corporation, Redmond, Washington, USA) and GraphPad Prism 9 (Version 9.1.1, GraphPad Software Inc., https://www.graphpad.com/scientific-software/prism/ California, USA). For distribution analysis, the D’Agostino–Pearson normality test was used. For a normal distribution, comparisons were made using a paired t-test. Otherwise, the Wilcoxon Sign Test was performed. MRI data was tested against reference value using a one-sample t-test. Correlation analysis using Pearson’s r test was performed using JASP^14^.

## Results

At time of the cluster outbreak SARS-CoV-2 infection was confirmed by virus specific RT-PCR in all eight athlethes. Sufficient viral RNA for whole genome sequencing was available in oropharyngeal swabs of five of them. Sequencing analysis showed high nucelic acid identity (> 99.9%) and revealed a lineage B.1.177.86 virus in all sequenced samples.

At time of the pre-season medical check-up presence of SARS-CoV-2 RNA was neither shown in the respiratory swabs nor in the mask swabs.

### Imaging

Echocardiography revealed no significant change in global longitudinal strain.

Cardiac resonance imaging showed native T2 values above the reference in the athletes with a history of SARS-COV-2 infection and the noninfected teammates.

Late gadolinium enhancement was present in two SARS-COV-2 positive athletes and none of the non-COVID-19 group (Table 1). No athlete reached all Lake-Louis criteria for the diagnosis of acute myocarditis.

One athlete exhibited a pericardial effusion in echocardiography and MRI, which declined in the subsequent examinations. This one was the only athlete with native T1 values above the reference and the highest T1- and T2-values.

### Spirometry

Table 2 summarizes the spirometric data. SARS-CoV-2 infection had no impact on pulmonary parameters under resting conditions. Interestingly, the non-COVID group showed a significant decrease in peak expiratory flow (PEF) and a significant increase in maximal expiratory flow at 25% of the vital capacity (MEF25).

**Table 2:** Results of spirometry and incremental cardiopulmonary exercise test (CPET).

	COVID-19 t0	COVID-19 t1	*p* value	Non-COVID t0	Non-COVID t1	*p* value
Spirometry	Mean (SD)	Mean (SD)		Mean (SD)	Mean (SD)	ns
FVC, l	6.5 (0.7)	6.5 (0.6)	ns	5.6 (2.5)	5.7 (2.6)	ns
FEV1, l/s	5.1 (0.5)	5.1 (0.5)	ns	4.1 (1.8)	4.3 (1.9)	ns
PEF, l/s	11.5 (1.5)	10.7 (0.8)	ns	9.3 (3.9)	8.6 (3.9)	0.036
MEF 25, l/s	2.2 (1.0)	2.1 (0.7)	ns	1.8 (0.5)	2.0 (0.7)	0.013
**CPET**						
Rest						
HR, 1/min	70 (8.2)	78 (10.2)	ns	76 (4.7)	79 (6.3)	ns
VO2, ml/min	428 (76)	451 (76)	ns	491 (37)	511 (81)	ns
VE, l/min	13 (1.5)	14 (2.1)	ns	14 (1.4)	15 (2.1)	ns
VTex, l	0.7 (0.2)	0.8 (0.2)	ns	0.7 (0.0)	0.8 (0.1)	ns
O2-pulse, ml/HR	6.1 (0.7)	5.8 (0.7)	ns	6.4 (0.3)	6.6 (1.2)	ns
RER	0.8 (0.1)	0.9 (0.1)	0.044	0.8 (0.1)	0.8 (0.1)	ns
Maximum						
Pmax, Watt	309 (33)	293 (37)	0.006	286 (24)	285 (23)	ns
HRmax, 1/min	183 (14)	190 (15)	0.038	187 (3.3)	183 (10)	ns
VO2max, ml/min	4082 (520)	3790 (513)	0.030	4222 (427)	3911 (46)	ns
VEmax, l/min	134 (15)	115 (17)	0.013	144 (44)	140 (22)	ns
VTex, l	2.9 (0.4)	2.8 (0.4)	ns	2.8 (0.2)	2.5 (0.2)	ns
O2-pulse, ml/HR	22.3 (2.6)	19.9 (1.7)	0.015	21.6 (2.2)	21.4 (1.1)	ns
RER	1.1 (0.1)	1.1 (0.1)	ns	1.1 (0.1)	1.1 (0.1)	ns

*FVC* Forced vital capacity, *FEV1* Forced expiratory volume in the first second, *PEF* Peak expiratory flow, *MEF* Maximal expiratory flow at 25% of the vital capacity, *HR* Heart rate, *VO2* Respiratory oxygen uptake, *VE* Respiratory minute volume, *VTex* Tidal volume, *RER* Respiratory exchange ratio, *Pmax* Maximal load, *ns* Not significant, *t0* Routine CPET during preparation phase in summer 2020, *t1* post-SARS-CoV-2 infection (COVID-19) in winter 2020 or during other cardiac outpatient diagnostics in winter 2020 (non-COVID).

### Cardiopulmonary exercise test (CPET)

**Figure 2:**
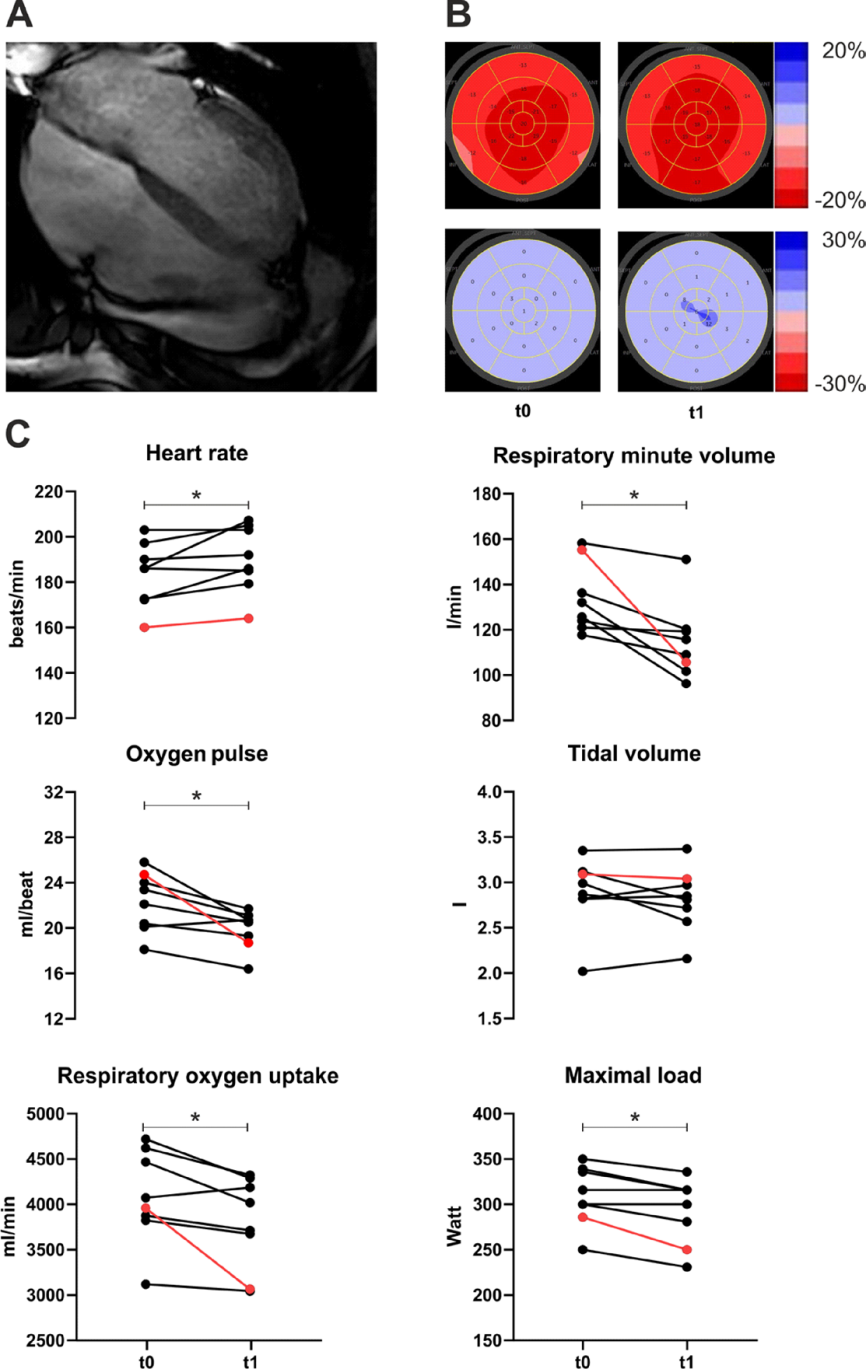
The athlete with the highest T1 values and pericardial effusion (red line) exhibited the greatest reduction in functional cardiopulmonary parameters. t0: Routine CPET during preparation phase in summer 2020 t1: post-SARS-CoV-2 infection (COVID-19) in winter 2020 of athletes with a history of SARS-CoV-2 infection. (**A**) cardiac resonance imaging of athlete with pericardial effusion. (**B**) longitudinal strain (red) and post systolic shortening index (blue) at t0 and t1. (**C**) key spiroergometric parameters at maximal load. The athlete with the highest T1-mapping values in magnetic resonance imaging and with pericardial effusion during the infection is highlighted in red. *: *p* < 0.05.

**Figure 3:**
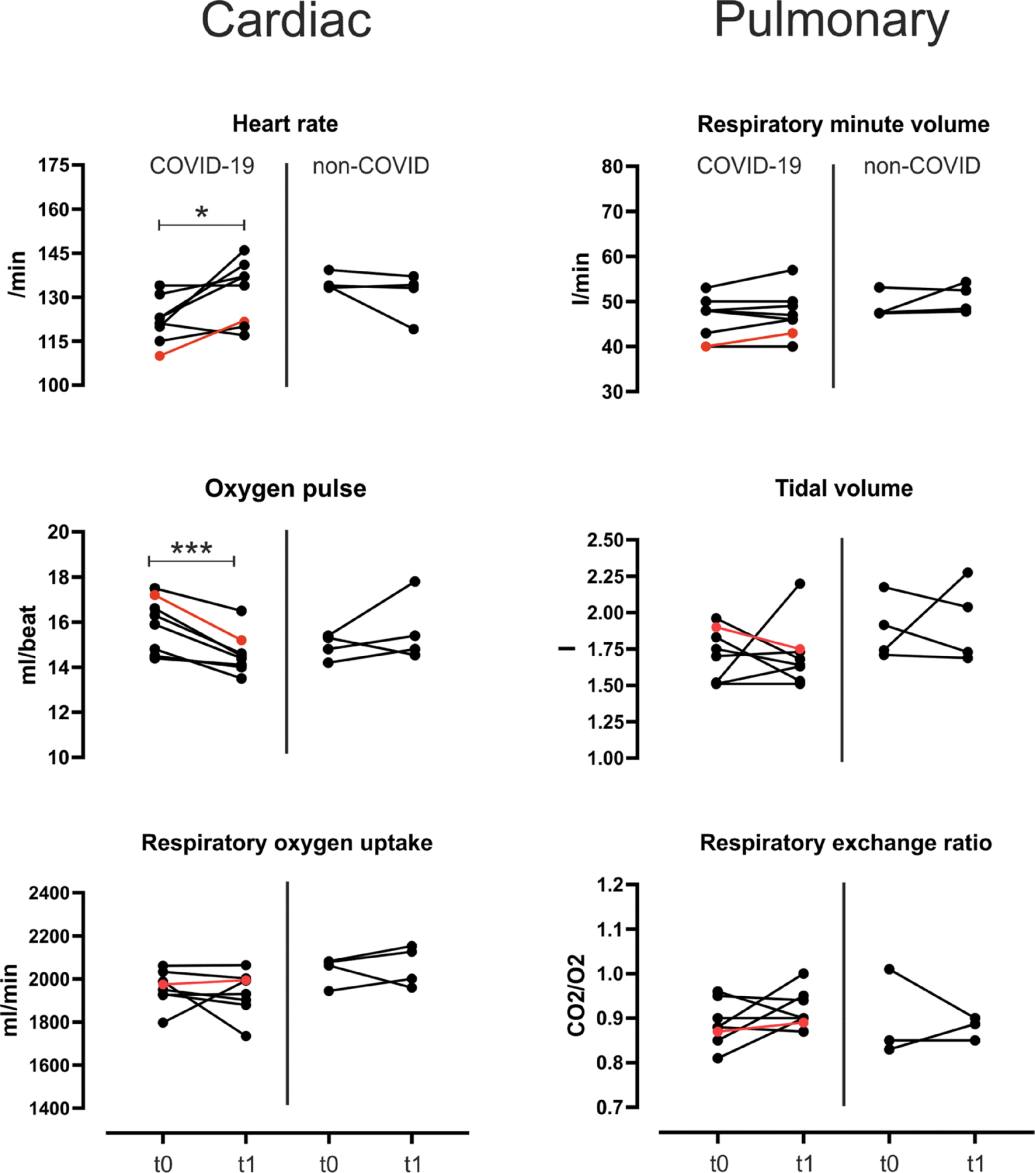
Mean values of cardiac and pulmonary parameters under moderate intensity in a CPET (100–200 Watt) in athletes with or without a history of SARS-CoV-2 infection. t0: Routine CPET during preparation phase in summer 2020 t1: post-SARS-CoV-2 infection (COVID-19) in winter 2020 or during other cardiac outpatient diagnostics in winter 2020 (non-COVID). The athlete with the highest T1-mapping values in magnetic resonance imaging and with pericardial effusion during the infection is highlighted in red. *: *p* < 0.05; ***: *p* < 0.001.

Results of CPET are shown in Table 2 and Figs. 2 and 3. SARS-CoV-2 infection did not affect cardiac or pulmonary parameters under resting conditions compared to the preparation phase in summer 2020 (t0). Athletes achieved maximal exertion (RER 1.1 vs. 1.1) in the preparation phase as well as after SARS-CoV-2 infection, while the maximum load was reduced significantly by 16 W (− 5.3%, *p* = 0.006). Maximal heart rate was seven beats/min (+ 3.7%, *p* = 0.038) higher after SARS-CoV-2 infection than pretest. The oxygen pulse was significantly reduced by 2.4 ml/beat (− 10.4%, *p* = 0.015). Respiratory oxygen uptake significantly decreased during the period by − 292 ml/min (− 7.0%, *p* = 0.03). These changes were not present in the non-COVID group. Pulmonary parameters showed only a significant reduction in respiratory minute volume by 18.9 l/min (− 13.8%, *p* = 0.013). One athlete showed a T-inversion under stress which declined completely in following examinations.

Notable, the athlete who had pericardial effusion and high T1- and T2- values in magnetic resonance imaging also had the most significant reduction in critical parameters of CPET (Fig. 2).

The effect of SARS-CoV-2 infection on cardiopulmonary parameters under moderate intensity (100–200 W) is shown in Fig. 3. The moderate-intensity corresponded to 52% and 53% of maximum respiratory oxygen uptake for COVID-19 and non-COVID groups. At this intensity level, significant changes in heart rate (+ 7.9%, *p* = 0.028) and O2-pulse (− 8.2%, *p* = 0.0008) were only observed in athletes with a history of COVID-19. No other parameters were changed significantly. The non-COVID group showed no significant changes during this period.

### Correlation analysis

Overall reduction in maximal load was correlated with maximum respiratory minute volume (R = 0.914, *p* < 0.001), maximum respiratory oxygen uptake (R = 0.838, *p* < 0.001), and the number of single cardiac segments above reference value in T1-mapping (R = 0.669, *p* = 0.017). This number also correlated to the decrease in maximum respiratory oxygen uptake (R = 0.711, *p* = 0.010) and respiratory minute volume (R = 0.701, *p* = 0.01). Notably, the COVID-19 group’s increase in global longitudinal strain strongly correlates to the rise in maximal heart rate (R = 0.778, *p* = 0.023) but not to other functional changes. Neither increased values in T2-mapping nor the number of single cardiac segments above reference value in T2-mapping correlated with functional cardiopulmonary parameters.

### RT-PCR swab of CPET-masks

All masks were tested negative for SARS-CoV-2 RNA after CPET using RT-PCR standard diagnostic procedure.

## Discussion

The CPET showed a significant reduction of cardiopulmonary performance in elite athletes after SARS-CoV-2 infection. The affected athletes had a reduced maximal load, maximal oxygen uptake, a higher heart rate at comparable exertion, and a significantly reduced O2 pulse. Additionally, the respiratory minute volume as a parameter of pulmonary function was considerably reduced. The cardiac changes in heart rate and oxygen pulse were present at moderate intensities, while the pulmonary effects became evident at higher intensity levels.

In contrast to the functional data, the morphological data only showed minor signs of inflammation that did not reach the Lake-Louis criteria for the diagnosis of acute myocarditis. However, changes in the global longitudinal strain strongly correlated to increasing maximal heart rate. In cardiac magnetic resonance imaging, the number of segments with increased T1-values correlated to maximum respiratory oxygen uptake decrease. In literature, isolated higher T1-values in athletes are also described as a possible effect of a history of intensive training^15^. Notably, the athlete with pericardial effusion and highest T1- and T2-values also had the most significant change in crucial spiroergometric parameters. His global longitudinal strain was not changed significantly, but it must be noted that heart rate was different between both assessments (t0: 56/min vs. t1: 40/min). GLS and heart rate have been shown to be negatively correlated^16^, so that the unchanged GLS in combination with reduced heart rate could potentially represent an impairment of left ventricular deformation. It can be assumed that conventional echocardiographic parameters of left ventricular function such as left ventricular ejection fraction and GLS are not suitable to detect minor cardiac involvement due to myocarditis^17^. Alterations of circumferential strain and rotational LV deformation might be more sensitive to detect minor functional alterations in infectious diseases due to distinct morphological alterations detected by cardiac MRI^18,19^.

Sequencing showed identical virus strains in all analysed respiratory swabs demonstrating a cluster infection. This finding is consistent with the results of contact tracing. The observed individual differences in clinical outcomes are therefore not explained by specific viral lineages or a specific virus strain. At the time the B1.177 variant was responsible for around 10 percent of all cases in Germany^20^.

Risks for clinical personnel during CPET are not well evaluated. During our study, SARS-CoV-2 RNA was not shown in mask swab samples. This finding suggests that athletes who repeatedly tested negative for SARS-CoV-2 RNA in nasopharyngeal swabs do not pose an infectious risk for the examining staff.

The effect of infectious and especially viral disease on cardiopulmonary performance is not well characterized outside of manifest myocarditis^21,22^. While guidance for athletes with manifest myocarditis is available^23–25^, the effect of SARS-CoV-2 infection on athletes missing this definition can still be relevant for safety while returning to competition. Measuring individual performance restrictions and specific areas to target or take care of in training is a valuable tool to avoid overload and regain optimal performance.

One could think that the fact that after diagnosis of SARS-CoV-2 infection the athletes did not participate in an exercise of any kind and were instructed to keep their heart rate at low levels could have led to a general change of cardiopulmonary parameters in CPET^26^. But contrary to this, it has been shown that after training pauses with a duration of around two to three weeks, like in our COVID-19 group, the athletes are generally expected to have a maintained cardiopulmonary performance with specific adaptations^27–30^. Additionally, the non-COVID control group did not participate in structured team training and did not show any signs of the effects seen in the COVID-19 group.

A possible explanation for the observed changes could be the prevalence of thrombosis and pulmonary microembolisms in COVID-19^31–33^, potentially contributing to reduced oxygen uptake, lower O2-pulse, and higher heart rate. We did not see any clinical indications of thrombosis or pulmonary embolisms. However, this chain of events cannot be discarded with current evidence, and further evaluation by prospective studies is necessary. The early changes under moderate intensities of cardiac parameters while pulmonary changes did only occur at maximal load could indicate a more pronounced functional effect of SARS-CoV-2 infection on the heart. On the other hand, it is known that the cardiac reserve may generally be smaller than the pulmonary reserve in elite athletes^34^. This higher pulmonary reserve could conceal pulmonary restrictions at moderate intensities.

The findings of this study identify spiroergometry as a valuable addition to imaging studies post COVID^4^. We screened athletes without myocarditis in the cardiac MRI using incremental cardiopulmonary exertion tests. Spiroergometry identified relevant functional impairment in nearly all athletes of our homogenous group of elite handball players. This, for the first time, shows the need for additional diagnostic measures to ensure a safe and optimal return to training.

Limitations of the study include the low number of evaluated athletes and the retrospective non-blinded analysis. However, the study group is highly homogeneous in terms of type of training and training status, patient characteristics and time of infection and representative of elite athletes. The functional and imaging measurements are highly standardized, and the parameters are well established from regular examinations in athletes at our institution.

## Conclusion

SARS-CoV2 infection caused functional impairment of cardiopulmonary performance in elite athletes. It seems reasonable and safe to screen athletes after SARS-CoV2 infection with spiroergometry to detect performance limitations. The knowledge of individual limitations and specific areas of higher or lower restriction can help to tailor training to individual needs for optimal effects in regaining performance.
